# Using ChatGPT for Clinical Practice and Medical Education: Cross-Sectional Survey of Medical Students’ and Physicians’ Perceptions

**DOI:** 10.2196/50658

**Published:** 2023-12-22

**Authors:** Pasin Tangadulrat, Supinya Sono, Boonsin Tangtrakulwanich

**Affiliations:** 1 Department of Orthopedics Faculty of Medicine Prince of Songkla University Hatyai Thailand; 2 Division of Family and Preventive Medicine Faculty of Medicine Prince of Songkla University Hatyai Thailand

**Keywords:** ChatGPT, AI, artificial intelligence, medical education, medical students, student, students, intern, interns, resident, residents, knee osteoarthritis, survey, surveys, questionnaire, questionnaires, chatbot, chatbots, conversational agent, conversational agents, attitude, attitudes, opinion, opinions, perception, perceptions, perspective, perspectives, acceptance

## Abstract

**Background:**

ChatGPT is a well-known large language model–based chatbot. It could be used in the medical field in many aspects. However, some physicians are still unfamiliar with ChatGPT and are concerned about its benefits and risks.

**Objective:**

We aim to evaluate the perception of physicians and medical students toward using ChatGPT in the medical field.

**Methods:**

A web-based questionnaire was sent to medical students, interns, residents, and attending staff with questions regarding their perception toward using ChatGPT in clinical practice and medical education. Participants were also asked to rate their perception of ChatGPT’s generated response about knee osteoarthritis.

**Results:**

Participants included 124 medical students, 46 interns, 37 residents, and 32 attending staff. After reading ChatGPT’s response, 132 of the 239 (55.2%) participants had a positive rating about using ChatGPT for clinical practice. The proportion of positive answers was significantly lower in graduated physicians (48/115, 42%) compared with medical students (84/124, 68%; *P*<.001). Participants listed a lack of a patient-specific treatment plan, updated evidence, and a language barrier as ChatGPT’s pitfalls. Regarding using ChatGPT for medical education, the proportion of positive responses was also significantly lower in graduate physicians (71/115, 62%) compared to medical students (103/124, 83.1%; *P*<.001). Participants were concerned that ChatGPT’s response was too superficial, might lack scientific evidence, and might need expert verification.

**Conclusions:**

Medical students generally had a positive perception of using ChatGPT for guiding treatment and medical education, whereas graduated doctors were more cautious in this regard. Nonetheless, both medical students and graduated doctors positively perceived using ChatGPT for creating patient educational materials.

## Introduction

Artificial intelligence (AI) is a new technology that has changed various industries, including medicine. AI refers to the development of computer systems capable of performing complex tasks that normally require human intelligence, such as understanding conversation, recognizing patterns or images, and making decisions. Traditionally, AI in medicine was used in areas such as medical imaging, diagnostics tests, and prediction tools. However, it evolved and became involved in other aspects of the medical field, for example, helping physicians gather patient data before the visit [1].

One of the most remarkable developments in AI is the advancement of large language models and natural language processing, which aim to facilitate the automatic analysis of language, mimicking human language understanding. ChatGPT is an application built based on large language models, namely, GPT-3.5 or GPT-4. This newly developed AI technology enables users to engage in interactive conversations and receive humanlike responses, thereby creating a more dynamic and engaging user experience [2]. ChatGPT fascinates many people in a variety of fields. In the medical field, it has been used to help write manuscripts [3-5]. However, researchers were still concerned about the contents’ ethical consideration and validity [6]. Many researchers have also evaluated ChatGPT for medical education, such as taking examinations and comparing the results to medical students [7-11]. The use of ChatGPT to help in the patient care process has also been reported [12,13].

The potential of using AI in the medical field, especially orthopedics, is promising. For example, deep learning AI has been used for detecting and classifying many orthopedic conditions, such as degenerative spinal conditions, rotator cuff injury, and implant loosening [14-16]. ChatGPT itself has been tested with the American Board of Orthopaedic Surgery Examination, but it cannot pass the exam [17]. One of the challenges encountered in medical practice is the high volume of patients, which may sometimes prevent physicians from providing detailed information to patients. Given that ChatGPT is a language model focused on communication, it could help provide appropriate treatment plans and patient education.

Therefore, we aim to investigate how medical students and practicing doctors perceive the use of ChatGPT in clinical settings and medical education. Additionally, we will explore whether there are differences in perception between medical students and doctors at various levels of experience regarding ChatGPT’s responses to a clinical question. We hypothesized that different levels of clinical experience would change participants’ perceptions of ChatGPT.

## Methods

### Ethical Considerations

This study was approved by the institutional review board (REC.66-125-11-1) at the Faculty of Medicine, Prince of Songkla University.

### Study Design

This was a cross-sectional study investigating the perceptions of medical students, interns, residents, and attending staff toward using an AI chatbot (ChatGPT) in clinical practice and medical education. Specifically, we asked participants to rate their opinions on the ChatGPT-generated treatment plan and advice using knee osteoarthritis as an example.

### Instrument

We developed a web-based questionnaire. The first part inquired about participants’ demographic data, including age, sex, and status. The second part explored participants’ general experience and perception toward using an AI system in medicine. The responses for the second and third parts used a Likert-scale system with five levels: strongly agree, agree, neither agree nor disagree, disagree, and strongly disagree.

The third part of the questionnaire explored the perception of the AI-generated response to a clinical question. We first gave ChatGPT (version 3.5) a question prompt: “Please act as a doctor and give me general knowledge, natural history and detailed treatment plan for a 65-year-old woman with knee osteoarthritis.” The response was shown in a questionnaire. We then asked participants to rate their perception of ChatGPT’s response validity, clinical reasoning, clinical application, and use as a patient education tool. Participants were asked if they could provide a better response than ChatGPT, and lastly, participants were asked to rate their perception of using ChatGPT’s response for medical education. In addition, we included open-ended questions for participants to express their opinions about the potential benefits and pitfalls of using ChatGPT for clinical practice and medical education.

A pilot test using a developed questionnaire was performed with 20 participants as the pilot group. The Cronbach α for internal consistency was .86.

### Participant Recruitment

The study was set in a university-affiliated teaching hospital. We recruited two groups of participants. The first group consisted of fifth-year medical students who had completed an orthopedics rotation. The second comprised graduated physicians of various levels, including interns, family medicine and orthopedic residents, and family medicine and orthopedic attendings. The questionnaire’s link was emailed according to the email list registered with the hospital.

### Data Analysis

All participants’ responses were exported as an Excel file (Microsoft Corporation) from the Google Form website. It was then imported and analyzed using the R program (version 4.2.3; R Foundation for Statistical Computing). Strongly agree and agree responses were grouped as a positive perception. Neither agree nor disagree responses were categorized as a neutral perception. Disagree and strongly disagree were grouped as a negative perception. Answers to the open-ended question were reviewed and discussed between investigators. Data distribution patterns were examined by histogram and Shapiro-Wilk test. Normally distributed continuous data were presented as means (SDs) and tested with an independent *t* test. Nonnormally distributed continuous variables were presented as medians (IQRs) and were tested with the Mann-Whitney *U* test. Categorical data were presented with count and percentage and tested with the Fisher exact probability test. Statistical significance was set at *P*<.05.

## Results

### Overview

We sent out 350 questionnaires and received 239 (68.2%) responses. A total of 124 of 185 (67%) medical students, 46 of 78 (59%) interns, 37 of 43 (86%) residents, and 32 of 44 (73%) attending staff responded. The median age of medical students, internists, residents, and attending staff were 23 (IQR 22-24), 25 (IQR 25-26), 29 (IQR 27-31), and 38 (IQR 35-47) years, respectively. Of the 239 respondents, 132 (55%) were female. Female respondents made up 79 of 124 (64%) medical students, 24 of 46 (52%) interns, 16 of 37 (43%) residents, and 13 of 32 (41%) attending staff.

Only 9 of the 239 (4%) respondents stated that they did not know about the concept of AI. When asked whether they used AI in their daily life, we found that 113 (47%) respondents rarely used it. Respondents who answered that they often used AI and who answered that they sometimes used AI were equal (n=39, 16%). Of the 239 respondents, 28 (12%) never used AI, and only 20 (8%) used AI regularly.

We specified the question further and inquired about the experience using an AI chatbot or ChatGPT in the medical field. Of the 239 respondents, 158 (66.1%) had never heard of AI in medicine or heard of it but never used it (Table 1). Even though there was a higher percentage of attending staff (13/32, 41%) and residents (10/37, 27%) who had never heard of AI chatbots or ChatGPT compared to interns (10/46, 22%) and medical students (18/124, 15%), the proportion of answers tested by Fisher exact test did not differ significantly between groups (*P*=.07).

**Table 1:** What is your experience using an AI chatbot or ChatGPT in the medical field?

	Use regularly, n (%)	Use sometimes, n (%)	Use rarely, n (%)	Heard of it but never use, n (%)	Never heard of it, n (%)
Medical student (n=124)	5 (4.0)	15 (12.1)	27 (21.8)	59 (47.6)	18 (14.5)
Intern (n=46)	2 (4.4)	7 (15.2)	8 (17.4)	19 (41.3)	10 (21.7)
Resident (n=37)	5 (13.5)	1 (2.7)	4 (10.8)	17 (46.0)	10 (27.0)
Staff (n=32)	1 (3.1)	2 (6.3)	4 (12.5)	12 (37.5)	13 (40.6)

Next, we evaluated respondents’ perceptions toward AI chatbots or ChatGPT use in clinical settings (part A of Multimedia Appendix 1). We found that a lower proportion of attending staff (16/32, 50%) and residents (20/37, 54%) had a positive perception toward the use of ChatGPT for clinical practice when compared to medical students (94/124, 76%) and interns (32/46, 70%). The difference between groups did not reach statistical significance (Fisher exact test *P*=.06). One attending who disagreed with using ChatGPT for clinical practice commented that patients prefer human interaction over a computer program. When asked whether ChatGPT could benefit medical education, most respondents had a positive perception (part B of Multimedia Appendix 1), with no significant difference between groups (*P*=.46).

Participants were asked to rate whether they agreed with the statement regarding the response from ChatGPT about treatment and patient education for knee osteoarthritis. We found that most participants agreed that the response from ChatGPT was valid and well reasoned (part A in Multimedia Appendix 2). The proportion of responses did not differ significantly between medical students, interns, residents, and attending staff (Fisher exact test *P*=.24). However, when asked whether they agreed that the responses were useful for clinical application, there was a statistical difference between the responses of each group (Fisher exact test *P*<.001). While medical students mostly agreed that it could be used in clinical practice, some attending staff, residents, and interns disagreed (part B in Multimedia Appendix 2). The result shows that some participants changed their minds after reading ChatGPT’s response. Of the 162 participants who felt positive toward using ChatGPT for patient care (part A of Multimedia Appendix 1), only 99 (61%) kept the same answer, while 54 (33%) changed to neutral and 9 (6%) changed to negative (part B in Multimedia Appendix 2).

Most participants agreed that the response from ChatGPT could be used to make educational media for patients (part C in Multimedia Appendix 2). The answer did not differ significantly between groups (Fisher exact test *P*=.83). When asked whether the participant could give a better treatment plan and patient education compared to the response from ChatGPT, we found a significant difference in answers between groups (Fisher exact test *P*<.001). While most medical students neither agreed nor disagreed with the statement, most residents and attending staff felt they could formulate a better treatment plan and give better advice (part D in Multimedia Appendix 2). Interestingly, some interns even rated ChatGPT’s response better than theirs. They explained that they could not provide advice as comprehensive as ChatGPT due to the time limit for each patient visit.

Lastly, we asked if the participants agreed that the responses from ChatGPT could be used as educational materials for medical students. Most medical students and residents agreed with the statement, but only about half of the attending staff and interns agreed. Of the 32 attending staff, 4 (13%) disagreed with the statement (part E in Multimedia Appendix 2). The proportional difference in answers between participant groups was statistically significant by Fisher exact test (*P*<.001).

A total of 32 participants gave additional comments about ChatGPT use for clinical practice and medical education. These responses could be categorized as the potential benefits, limitations, and pitfalls of using ChatGPT.

### ChatGPT in Medical Education

#### Potential Benefits

Some medical students commented that the responses generated could be used to prepare for the objective structured clinical examination (OSCE), especially for the question that asks the student to give advice and a general treatment plan. Some attending staff and residents stated that it could be used to review and conceptualize the understanding of each disease.

#### Limitations and Pitfalls

Medical students did not give any comment regarding limitations. However, there were many concerns from attending staff, residents, and interns. Many respondents felt that the response generated by ChatGPT was superficial and too general. They believed that medical students should pursue a deeper understanding of the disease.

Several participants also commented that the knowledge, even though it is valid, may lack proper supporting scientific evidence, and medical students should learn to acquire and evaluate new knowledge from standard and trustworthy sources. The reliability of the answers was another concerning point. Respondents still doubted whether ChatGPT could produce a valid response for all diseases. One attending staff who disagreed about using ChatGPT for medical education stated that the lack of content verification by experts was another major concern.

### ChatGPT in Clinical Practice

#### Potential Benefits

The majority of respondents agreed that the answers from ChatGPT are suitable for general treatment planning. Many also stated that the answer could be used as a template for making patient education media.

#### Limitations and Pitfalls

Respondents raised several limitations. First, the treatment plan was too generalized and may not be suitable for different patients. They also stated that physicians need to make an individualized treatment plan for each patient according to many factors, such as disease severity, lifestyle, and patient expectations. Second, respondents were also concerned about whether the AI could provide up-to-date treatment information and suggested that physicians must regularly update their knowledge from trustworthy sources. Third, many worried about the language barrier. ChatGPT was created using English as the primary language. The meaning and correctness must be re-evaluated when the information is translated to make patient education media. Lastly, almost all respondents were concerned about data bias. ChatGPT was trained from massive internet data; however, the sources were not always from an appropriate scientific database. Therefore, the resulting answer may not be correct.

## Discussion

This study reflected how medical students and various levels of physicians felt about medical answers from ChatGPT and its applications. We found that participants with different clinical experience levels had different perceptions toward ChatGPT’s use for clinical practice and medical education. Medical students generally had a positive perception, while practicing physicians were more neutral.

For clinical practice, a higher proportion of attending staff and residents disagreed with using ChatGPT. While medical students were satisfied with responses that followed textbooks and sounded authentic, more experienced physicians could detect the pitfalls of the responses. They had shared their concerns, which had both supporting and conflicting literature.

The first concern was the lack of patient-specific treatment plans. ChatGPT seemed to provide accurate and reproducible advice for general knowledge. For example, bariatric surgeons rated responses of ChatGPT as “comprehensive” for 86.8% of the questions asked [18]. Gastroenterologists also rated ChatGPT’s response to common patient questions with a score of 3.9 (SD 0.8), 3.9 (SD 0.9), and 3.3 (SD 0.9) out of 5 for accuracy, clarity, and efficacy, respectively [12]. It could provide a well-structured and comprehensive response to common breast augmentation surgery questions [19]. The responses to common questions about retinal detachments were rated appropriate in 80%-90% of the questions asked [20]. However, patient-specific conditions should also be included in treatment planning. The most appropriate treatment method selection may need clinical reasoning and experience. Therefore, ChatGPT’s answer could be used as a general outline for treatment, but currently, it could not replace a physician’s clinical reasoning and judgment. If the model is further explicitly trained for some medical conditions, it might be able to provide more specific treatment recommendations.

Another concern about using ChatGPT in clinical practice was its evidence-based element. It seemed that ChatGPT gathered resources from reasonably reliable sources. For example, in responding to public health questions, 91% of the answers given were determined to be based on evidence [21]. However, there were reports of ChatGPT citing nonexistent publications when asked [22]. Data validity was another point of concern. Due to increasing numbers of publications and emerging predatory publishers, ChatGPT might have relied on references that it deemed valid but were, in fact, fraudulent. Therefore, physicians may still have advantages over AI because they can assess and choose the most valid, reliable, and up-to-date knowledge for their clinical practice.

Most participants agreed that ChatGPT could be used for patient education. Some research also supported this opinion. ChatGPT had the potential to be used as a diabetic educator [23]. It could also provide an effective diet plan for people with food allergies, albeit with minor errors [13]. ChatGPT correctly answered 61% of basic public medical consultations, but only 39% of questions asked by health care personnel were correctly answered [24]. It seemed that for general medical questions, ChatGPT could generate appropriate advice. However, for more specific topics, the development of a dedicated chatbot might be more beneficial. For example, the SnehAI chatbot was developed to educate adolescents in India about sexual health and showed promising results [25]. Another chatbot, “VIRA,” was created to communicate and ensure COVID-19 vaccine safety with young adults and minority populations [26].

In medical education, ChatGPT could be used in various aspects [27]. Using ChatGPT for preparing for OSCE and other exams was mentioned by participants and in the literature [28]. For OSCE, it could help by generating example scenarios, suggesting a proper physical examination, and giving appropriate medical advice. Surprisingly, it could score even higher than humans for a virtual OSCE in obstetrics and gynecology [29]. However, it should be noted that ChatGPT responses were compared to only two human candidates and might not represent the whole picture. For multiple-choice question examinations, ChatGPT could answer some questions correctly and give explanations with acceptable insights and reasoning. However, the results of using ChatGPT were quite varied, from passing the exam to failing some [7,8,10,30-33]. When explored in detail, the passing score of ChatGPT in most tests was at average or slightly above minimal passing level. Therefore, it supported the fact that many attending staff and residents felt that the response by ChatGPT was superficial and did not show a deep understanding of the topic. For more advanced examination levels, such as resident-level examinations, ChatGPT performed more poorly [7,34,35]. For example, ChatGPT’s score in the plastic surgery in-training examination was ranked at the 49th percentile compared with first-year residents but significantly worse than fifth- and sixth-year residents at the zeroth percentile [9]. However, more recent research using an updated GPT-4 model capable of advanced reasoning and complex problem-solving showed remarkable results, and the GPT-4 model consistently outperforms GPT-3.5. GPT-4 was able to pass the Peruvian National Licensing Examination, the Japanese Medical Licensing Examination, German medical state examinations, and the Family Medicine Residency Progress Test with exceptional scores [11,36-38].

Our study tried to gather information from different levels of students and physicians and contrasted their results. We found that less experienced medical students might overlook some potential pitfalls of using ChatGPT in clinical practice and medical education. Even though there were many benefits of using ChatGPT, medical teachers needed to be aware of the risks and warn their medical students accordingly.

The limitation of our study was that we used only one scenario of knee osteoarthritis. If there were more scenarios of other diseases, the perception might differ; however, we felt that knee osteoarthritis was a good representation of a condition commonly encountered by various levels of physicians and would generate a diverse response. Moreover, ChatGPT has been known to answer according to the prompt and may change its answer depending on how the question was asked. In our study, the question contained the “General knowledge” word, which might affect how the respondent rates the answer. The participants also came from one center, which could limit the generalizability of the results. Additionally, the response rate of 68.2% might indicate the selection bias toward people who were already interested in AI, therefore, boosting the positive perception toward ChatGPT. Furthermore, besides the limited representativeness of doctors and medical students within the survey setting, the omission of patient perspectives neglected the input of arguably the most crucial stakeholder in health care. Lastly, the latest ChatGPT model is GPT-4, which is more advanced and may be able to provide more detailed responses. However, the superiority of ChatGPT-4 compared to ChatGPT-3.5 has mainly been proven in a scenario of multiple-choice examinations.

In conclusion, medical students generally had a positive perception of using ChatGPT for guiding treatment and medical education, whereas graduated doctors were more cautious in this regard. Nonetheless, both medical students and graduated doctors positively perceived using ChatGPT for creating patient educational materials.

## Appendix

Multimedia Appendix 1 (A) Perceptions toward using artificial intelligence (AI) chatbot for patient care. (B) Perception toward AI for medical education.

Multimedia Appendix 2 (A) Perception toward validity and clinical reasoning of ChatGPT's response. (B) Perception toward using ChatGPT's response in clinical practice. (C) Perception toward using ChatGPT's response for patient education material. (D) Perception of self-advice compared to ChatGPT. (E) Perception toward using ChatGPT's response for medical education.

## Abbreviations

AI: artificial intelligence
OSCE: objective structured clinical examination
