# Supplementary figures and images for: Using ChatGPT for Clinical Practice and Medical Education: Cross-Sectional Survey of Medical Students’ and Physicians’ Perceptions
Source: JMIR Med Educ. 2023 Dec 22;9:e50658. doi: 10.2196/50658 (PMC10770783; doi:10.2196/50658)

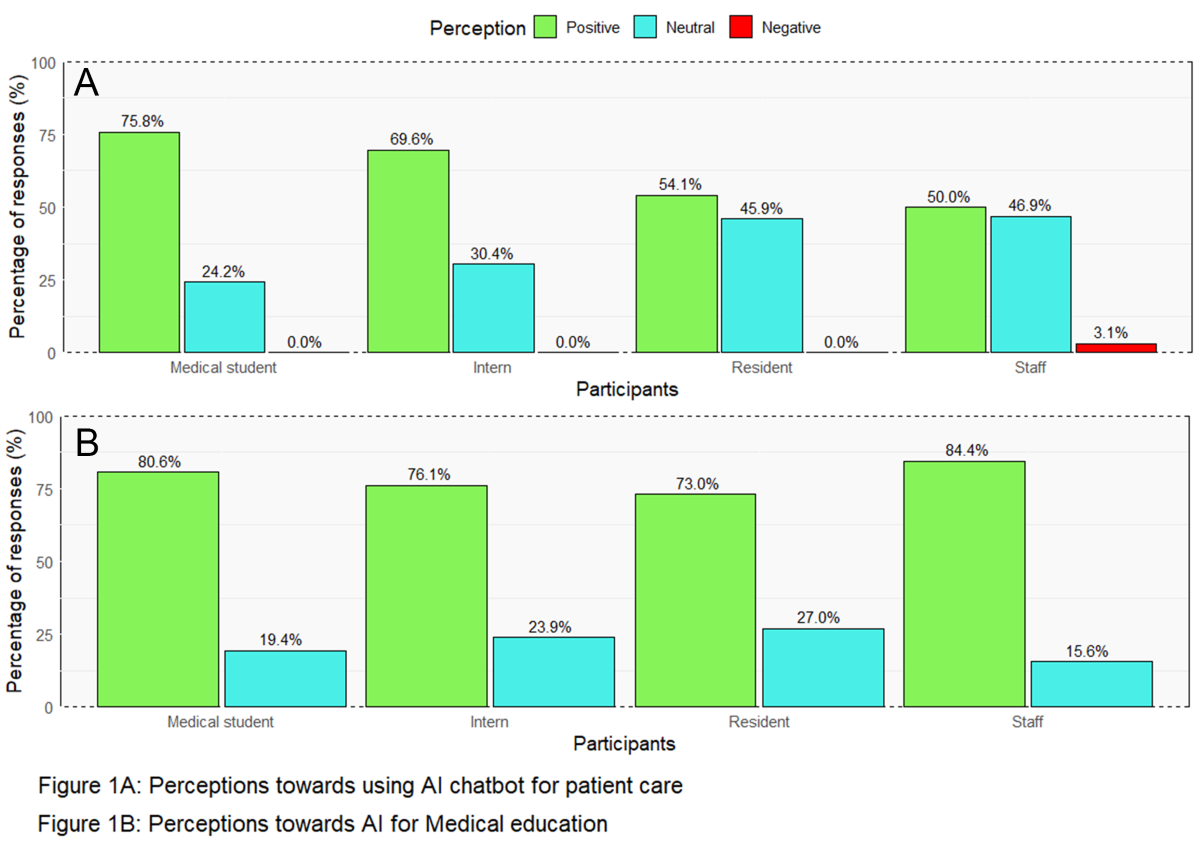

Supplement: Multimedia Appendix 1 [file mededu_v9i1e50658_app1.png]

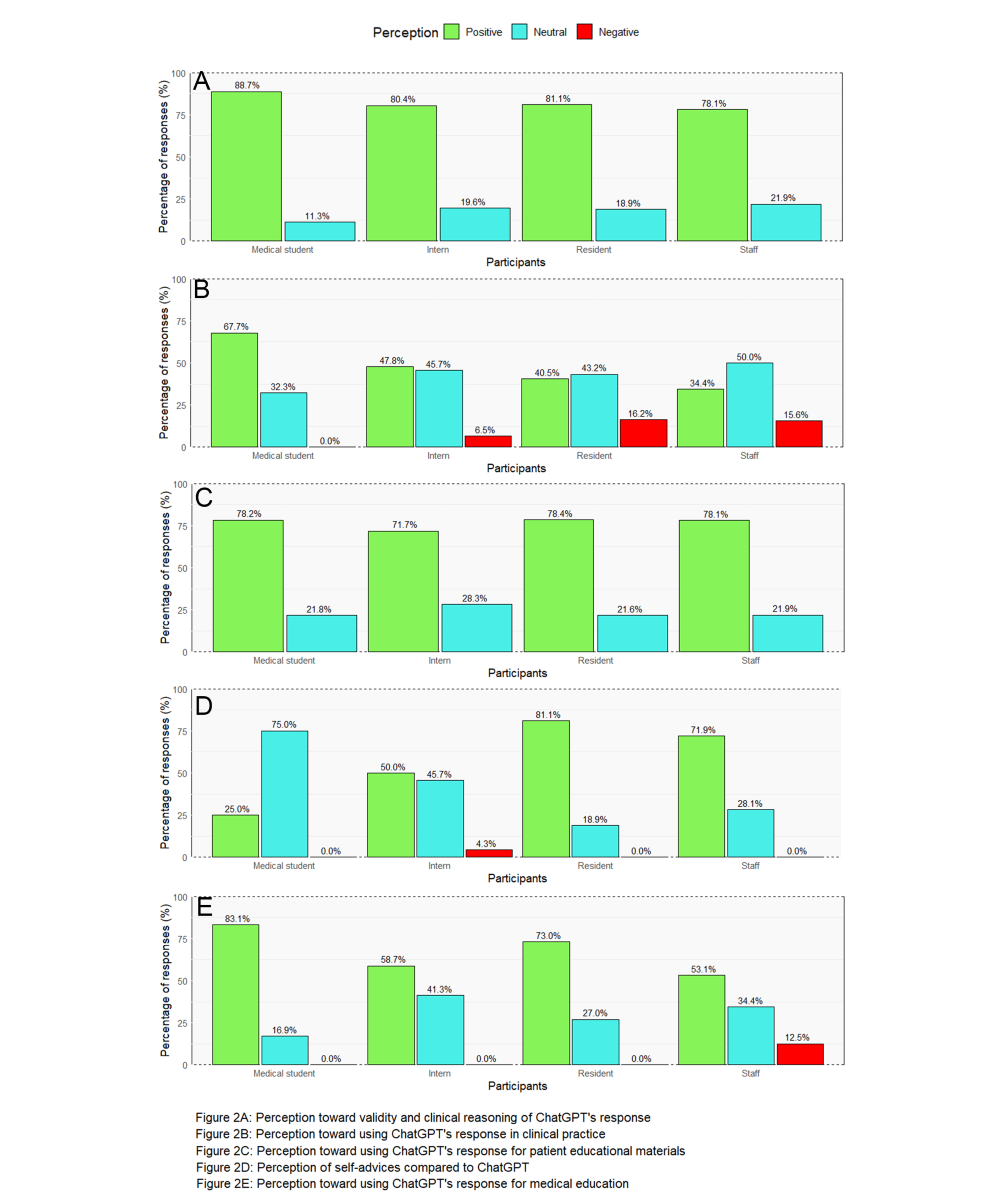

Supplement: Multimedia Appendix 2 [file mededu_v9i1e50658_app2.png]
